# Ca^2+^ Cycling Impairment in Heart Failure Is Exacerbated by Fibrosis: Insights Gained From Mechanistic Simulations

**DOI:** 10.3389/fphys.2018.01194

**Published:** 2018-08-23

**Authors:** Maria T. Mora, Jose M. Ferrero, Juan F. Gomez, Eric A. Sobie, Beatriz Trenor

**Affiliations:** ^1^Centro de Investigación e Innovación en Bioingeniería, Universitat Politècnica de València, Valencia, Spain; ^2^Department of Pharmacological Sciences, Icahn School of Medicine at Mount Sinai, New York, NY, United States

**Keywords:** calcium handling, heart failure, fibrosis, sensitivity analysis, electrophysiology

## Abstract

Heart failure (HF) is characterized by altered Ca^2+^ cycling, resulting in cardiac contractile dysfunction. Failing myocytes undergo electrophysiological remodeling, which is known to be the main cause of abnormal Ca^2+^ homeostasis. However, structural remodeling, specifically proliferating fibroblasts coupled to myocytes in the failing heart, could also contribute to Ca^2+^ cycling impairment. The goal of the present study was to systematically analyze the mechanisms by which myocyte–fibroblast coupling could affect Ca^2+^ dynamics in normal conditions and in HF. Simulations of healthy and failing human myocytes were performed using established mathematical models, and cells were either isolated or coupled to fibroblasts. Univariate and multivariate sensitivity analyses were performed to quantify effects of ion transport pathways on biomarkers computed from intracellular [Ca^2+^] waveforms. Variability in ion channels and pumps was imposed and populations of models were analyzed to determine effects on Ca^2+^ dynamics. Our results suggest that both univariate and multivariate sensitivity analyses are valuable methodologies to shed light into the ionic mechanisms underlying Ca^2+^ impairment in HF, although differences between the two methodologies are observed at high parameter variability. These can result from either the fact that multivariate analyses take into account ion channels or non-linear effects of ion transport pathways on Ca^2+^ dynamics. Coupling either healthy or failing myocytes to fibroblasts decreased Ca^2+^ transients due to an indirect sink effect on action potential (AP) and thus on Ca^2+^ related currents. Simulations that investigated restoration of normal physiology in failing myocytes showed that Ca^2+^ cycling can be normalized by increasing SERCA and L-type Ca^2+^ current activity while decreasing Na^+^–Ca^2+^ exchange and SR Ca^2+^ leak. Changes required to normalize APs in failing myocytes depended on whether myocytes were coupled to fibroblasts. In conclusion, univariate and multivariate sensitivity analyses are helpful tools to understand how Ca^2+^ cycling is impaired in HF and how this can be exacerbated by coupling of myocytes to fibroblasts. The design of pharmacological actions to restore normal activity should take into account the degree of fibrosis in the failing heart.

## Introduction

Heart failure (HF) is a major public health problem worldwide (Savarese and Lund, 2017) and the development of appropriate therapies to manage HF requires an in-depth knowledge of this syndrome. HF is classified as HF with reduced ejection fraction (HFrEF) or HF with preserved ejection fraction (HFpEF) according to left ventricular systolic function and the type of remodeling (Fukuta and Little, 2007). In HFrEF, the heart is unable to pump blood efficiently due to a reduction in cardiac contractility after diverse cardiovascular diseases. The complexity of the excitation–contraction sequence and the multiscale problem can be approached through mathematical models, which significantly help to gain insight into the underlying mechanisms of cardiac dysfunction and guide future research lines (O’Hara et al., 2011; Trayanova and Chang, 2016).

Contractile dysfunction in HF has been associated with an altered Ca^2+^ handling in myocytes, since Ca^2+^ homeostasis is crucial for cell contraction and relaxation (Alpert et al., 2000; Bers, 2000). Failing myocytes present a diminished intracellular Ca^2+^ transient (CaT) with a slow rise time and a reduced rate of [Ca^2+^]_i_ removal that prolong CaT duration and elevate the diastolic intracellular Ca^2+^ level (Piacentino et al., 2003; Lou et al., 2012). Prolonged action potential duration (APD) and [Na^+^]_i_ increase are other of the hallmark electrophysiological abnormalities in HF and all of them result from ion channel remodeling in myocytes, i.e., changes in the expression and function of proteins involved in the electrical activity of cells (Gomez et al., 2014b).

A tissue-level hallmark of HF is increased fibrosis and proliferation of cardiac fibroblasts. Several *in vitro* studies and mathematical modeling studies have documented that electrical coupling between myocytes and fibroblasts will lead to changes in APD and intracellular Ca^2+^ (Zhan et al., 2014; Li et al., 2017). Experimental evidence suggesting the formation of gap junctions between myocytes and fibroblasts *in vitro* (Gaudesius et al., 2003) has focused researchers attention on the altered electrophysiological properties of myocytes due to this intercellular coupling to explain conduction abnormalities and reentries (Miragoli et al., 2006; Xie et al., 2009a; Majumder et al., 2012). We have already addressed, in a previous work, the consequences on electrical propagation in cardiac tissue under conditions of HF and fibrosis confirming the vulnerability to reentrant activity (Gomez et al., 2014a,b). While electrical changes, with a cellular origin in action potential (AP) properties, have been widely investigated in the heterocellular coupling (Nguyen et al., 2012; Sridhar et al., 2017), changes in Ca^2+^ dynamics have not been explored in depth. It is important, therefore, to understand the role of fibroblasts in the modulation of Ca^2+^ cycling and to progress in the management of HFrEF, improving mechanical contraction.

Therefore, the goal of the present study was to investigate with computational models the effects of fibroblasts on ion transport mechanisms that regulate Ca^2+^ handling in human failing cardiomyocytes. To understand the complex processes taking place in these cells, we made use of sensitivity analyses. Sensitivity calculation has been commonly used for its predictive value in determining electrophysiological properties with parameter variability (Romero et al., 2011; Trenor et al., 2012; Walmsley et al., 2013; Cummins et al., 2014; Mayourian et al., 2016). As univariate and multivariate sensitivity analyses are widely used (Romero et al., 2009; Sobie, 2009), a comparison of both approaches was an initial objective of this work. Inter-subject variability in electrophysiological properties was considered and reproduced by populations of models. Failing populations, with drug-induced alterations in addition to the natural variability, were useful to identify specific combinations of model parameters that could counteract the effects of HF remodeling and fibroblasts. Our results identify the main targets to improve Ca^2+^ dynamics under the pathological conditions explored, improving cardiac contraction recovery.

## Materials and Methods

### Cellular Models

All simulations were performed at the cellular level. To study the electrophysiological behavior of cardiac myocytes, we used the most complete undiseased human ventricular AP model, developed by O’Hara et al. (2011) (ORd model), which comprises 15 sarcolemmal currents, as shown in Eq. 1, known as fast Na^+^ current (I_Na_), late Na^+^ current (I_NaL_), transient outward K^+^ current (I_to_), L-type Ca^2+^ current (I_CaL_), Na^+^ current through the L-type channel (I_CaNa_), K^+^ current through the L-type channel (I_CaK_), rapid delayed rectifier K^+^ current (I_Kr_), slow delayed rectifier K^+^ current (I_Ks_), inward rectifier K^+^ current (I_K1_), Na^+^/Ca^2+^ exchange current (I_NCX_), Na^+^/ K^+^ ATPase current (I_NaK_), background currents (I_Nab_, I_Cab_, I_Kb_), and sarcolemmal Ca^2+^ pump current (I_pCa_). A detailed Ca^2+^ dynamics is also formulated in the model. Properties such as conductances determining ionic densities and membrane kinetics can be found in the original work (O’Hara et al., 2011). We introduced slight modifications in sodium current formulation, as reported in our previous work (Mora et al., 2017) and leading to ORdmm model, which can also be found in **Supplementary Table S1**. To reproduce HFrEF phenotype, specific parameters of the model were modified to represent the downregulation or upregulation of cellular proteins experimentally observed in failing cells. This electrophysiological remodeling involved different ion currents and Ca^2+^ fluxes and has already been described in previous studies of our group (Gomez et al., 2014b; Mora et al., 2017). Specifically, the time constant of inactivation of I_NaL_ (τ_hL_), I_NaL_ conductance, the maximal flux of I_NCX_ and SR Ca^2+^ leak (J_leak_), and the fraction of active binding sites of the Ca^+2^ calmodulin-dependent protein kinase II (CaMKa) were upregulated, while conductances of I_to_ and I_K1_, the maximal flux of I_NaK_, and Ca^2+^ uptake via SERCA pump (J_SERCA_), and SR Ca^2+^-dependence of the steady-state activation of ryanodine receptor release (K_rel,Ca_) were downregulated. Further details about values and experimental references can be found in **Supplementary Table S2**.

(1) $$\begin{array}{c} I_{\mathrm{ion}}=\;I_{\mathrm{Na}}{+I}_{\mathrm{NaL}}{+I}_{\mathrm{to}}{+I}_{\mathrm{CaL}}{+I}_{\mathrm{CaNa}}{+I}_{\mathrm{CaK}}{+I}_{\mathrm{Kr}}{+I}_{\mathrm{Ks}}+\;I_{K1}{+I}_{\mathrm{NaCa}}{+I}_{\mathrm{NaK}}{+I}_{\mathrm{Nab}}{+I}_{\mathrm{Cab}}{+I}_{\mathrm{Kb}}{+I}_{\mathrm{pCa}}\;\left(1\right) \end{array}$$

The increase in fibroblasts density due to aging and/or HF, and their interaction with myocytes were modeled using established cell–cell coupling equations. Fibroblasts were resistively coupled to one myocyte (Eq. 2), and a current (I_gap_) flows from one cell to the other through gap junctions, driven by the potential gradient between the myocyte (V_M_) and the fibroblast (V_F_) membrane potential and regulated by a junctional conductance (G_gap_).

(2) $$\begin{array}{c} I_{\mathrm{gap}\;}=\;G_{\mathrm{gap}\;}\cdot \;(V_{M}{-V}_{F})\;\left(2\right) \end{array}$$

The electrical activity of fibroblasts was formulated using an active fibroblast model (MacCannell et al., 2007). The fibroblast AP model incorporates four transmembrane currents: a time- and voltage-dependent delayed-rectifier K^+^ current (I_Kr_), an inward-rectifying K^+^ current (I_K1_), a Na^+^/K^+^ ATPase current (I_NaK_), and a background Na^+^ current (I_b,Na_). The membrane potential is governed by the following ordinary differential equation:

(3) $$\begin{array}{c} \frac{{\mathrm{dV}}_{F}}{\mathrm{dt}}\;=\;-\frac{1}{C_{F}}\;(I_{\mathrm{Kr}}\;+\;I_{K1}\;+\;I_{\mathrm{NaK}}\;+\;I_{b,\mathrm{Na}\;}-\;I_{\mathrm{gap}})\;\left(3\right) \end{array}$$

The electrotonic interaction with the myocyte is included in the term I_gap_. Regarding fibroblast properties, the membrane capacitance (C_F_) was set to 6.3 pF and the resting membrane potential (E_F_) had a value of -49.6 mV as in MacCannell et al. (2007). Similarly, the differential equation for the membrane potential of the myocyte, taking into account myocyte–fibroblast coupling, is as follows:

(4) $$\begin{array}{c} \frac{{\mathrm{dV}}_{M}}{\mathrm{dt}}=-\frac{1}{C_{M}}(I_{\mathrm{ion}}{+I}_{\mathrm{stim}}{+I}_{\mathrm{gap}})\;\left(4\right) \end{array}$$

Myocyte dimensions are bigger than fibroblasts, which is considered in membrane capacitance differences (C_M_ = 153.4 pF vs C_F_ = 6.3 pF). The value of G_gap_ was set to 3 nS for a single fibroblast, considered the nominal value in MacCannell et al. (2007) model. This value is within the range of 0.3–8 nS measured in cultured myocyte–fibroblast pairs (Rook et al., 1992). In our simulations, an elevated degree of fibrosis was considered by setting a myocyte–fibroblast ratio of 1:5, obtained by increasing G_gap_ fivefold (Mayourian et al., 2016; Zimik and Pandit, 2016). In the absence of fibrosis, G_gap_ is 0, and the myocyte is not coupled to fibroblasts.

Both types of myocytes, with and without electrophysiological HF remodeling, were coupled to the same number of fibroblasts with identical properties to simulate the effect of fibrosis on a single myocyte under different conditions. Four basic models were then considered: ORdmm model, ORdmm model with HF remodeling, ORdmm model with five coupled fibroblasts, and HF ORdmm model coupled to five fibroblasts.

### Measurement of Biomarkers

To evaluate the electrophysiological activity of myocytes, and particularly Ca^2+^ dynamics, the electrophysiological indicators chosen were APD from maximal upstroke to 90% of repolarization (APD_90_), calcium transient (CaT) duration from maximal upstroke to 80% of recovery (CaTD_80_), CaT rise time from 10 to 90% of upstroke (*t*_10-90_), and systolic and diastolic [Ca^2+^]_i_ values. All biomarkers were calculated under steady state conditions after application of 1000 stimuli at 1 Hz.

### Population of Models

The aforementioned models were used as the baseline to generate four populations of 300 different individuals. Inter-subject electrophysiological variability was represented by variating maximal ion current conductances with a scale factor, assuming that there are variations in the number of ion channels in the cell membrane between individuals (Muszkiewicz et al., 2016). The variation in these scale factors is also a way to simulate the effects of drugs on ion channels, as a simplified action of pharmacological compounds inhibiting or enhancing ion currents. We selected and modified 13 key variables of the model, accounting for maximal ionic conductances and fluxes of I_Na_, I_NaL_, I_to_, I_CaL_, I_Kr_, I_Ks_, I_K1_, I_NaK_, I_NCX_, the SR Ca^2+^ uptake via SERCA pump (J_SERCA_), the SR Ca^2+^ release flux via RyR (J_rel_), the SR Ca^2+^ leak (J_leak_), and I_Nab_. These parameters were varied with a scaling factor obtained from a log-normal distribution of standard deviation (σ) equal to 0.3. This led to a 95% (±2σ) of parameters varying between 55 and 182% of its control value, representing inter-individual variability and drug-induced effects. A standard deviation of 0.1 was also considered in different sets of populations.

In order to analyze how failing APs and CaTs could be restored to normal physiological ranges, larger populations of models were generated. Latin Hypercube Sampling (LHS) of parameters in the range of ±60% variation was used for this purpose. In this case, only the basic HF model (with and without coupled fibroblasts) was considered. We set limits for APD_90_ and systolic and diastolic [Ca^2+^]_i_ values, in which electrical myocyte activity was considered normal. These limits were obtained from the baseline models as APD_90normal_ ± (APD_90HF_ - APD_90normal_)/2 and after being compared to experimental studies (Drouin et al., 1998; Li et al., 1998; Pereon et al., 2000). **Table 1** shows the considered ranges.

**Table 1:** Biomarkers ranges for normal electrophysiological activity.

	Basic ORdmm	Normal range		Basic ORdmm HF
		Min	Max	
APD90 (ms)	290.6	240	341	371.8
systolic [Ca^2+^]_i_ (nM)	420	300	1000	190
diastolic [Ca^2+^]_i_ (nM)	80	50	200	100

### Sensitivity Analysis

Linear regression was employed to analyze biomarker sensitivities to electrophysiological variables. First, a univariate sensitivity analysis was conducted varying each parameter individually by a scaling factor of equal magnitude from the baseline models. The individual variation was set to ±60 or ±15% in different sensitivity analyses. This approach was based on our previous work (Mora et al., 2017).

Multivariate regression analyses were then performed using the generated populations of models. We related all the conductance scaling factors configurations (x) to each biomarker (y) with regression coefficients (b) that attempted to predict the indicators (Eq. 5). These coefficients were obtained by applying partial least squares (PLS) method to data as shown in Eq. 6 and after several transformations, such as log-transformations and z-score calculations (Sobie, 2009; Morotti et al., 2017). The obtained coefficients indicate the relative contribution of parameters variation in biomarker changes and can be considered as sensitivities. This methodology takes into account inter-variable effects that cannot be differentiated in the univariate analyses. Coefficients of determination (*R*^2^) indicated the predictive power of the simplified linear model, being *R*^2^ = 1 a good fit.

(5) $$\begin{array}{c} y_{\mathrm{predicted}}=\sum b\;\cdot \;x\;\left(5\right) \end{array}$$

(6) $$\begin{array}{c} B_{\mathrm{PLS}}=\;{\left(X^{T}X\right)}^{-1}\;\times \;X^{T}\times \;Y\;\left(6\right) \end{array}$$

Both types of sensitivity analyses were compared with normalized sensitivities to study the similarities and differences in parameter identification as the most important contributors to each electrophysiological characteristic. Normalized sensitivities were calculated as the ratio between each sensitivity and the maximum absolute value obtained for a particular biomarker.

The comparison between isolated and coupled myocytes was performed after adjusting the bar graphs to the standard deviation of the log-transformed biomarkers in N and HF conditions, to ensure equivalent percentages of change.

## Results

### Comparison of Univariate and Multivariate Sensitivity Analyses in Normal and Failing Conditions

**FIGURE 1:**
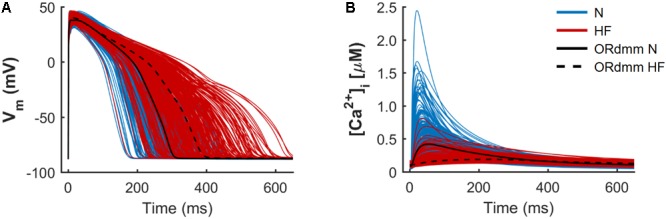
Population of normal (N, blue) and heart failure (HF, red) human ventricular cell models. Simulated action potentials **(A)** and intracellular Ca^2+^ transients **(B)** obtained from a multivariate set of ionic conductances (*n* = 300), using the baseline ORdmm model in N conditions (black solid line) and with HF ionic remodeling (black dashed line).

**Figure 1** illustrates two populations of models (*n* = 300) for endocardial human cells paced at 1 Hz, under normal (blue traces) and HF (red traces) conditions, with baseline models for the two respective populations indicated with black solid and dashed lines. The model population calibration process, meant to reproduce natural variability, generates a wide range of physiological AP waveforms and CaTs in both groups. This variability allows us to understand predicted drug effects by considering the HF cells with behavior most similar to normal cells, or vice versa. The electrophysiological remodeling applied to the basic ORdmm model to generate a baseline HF model reproduced the characteristic HF phenotype of prolonged APD and a slower Ca^2+^ dynamics with depressed systolic [Ca^2+^]_i_ and elevated diastolic levels. In the generated normal and failing populations, significant differences between both conditions are observed in APs and CaTs, although some traces overlap. To understand the variability within the populations and predict what alterations might cause HF cells to behave more like normal cells, we performed a sensitivity analysis.

**FIGURE 2:**
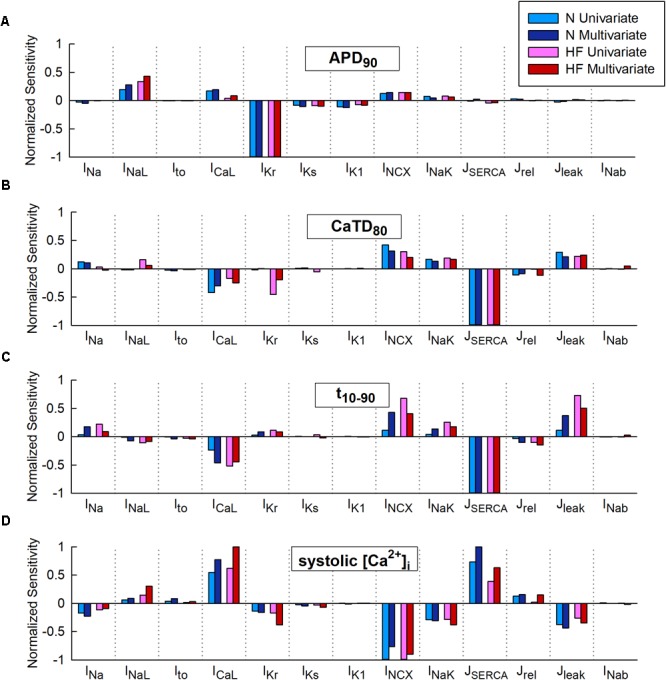
Comparison of univariate and multivariate relative sensitivities in normal (N) and heart failure (HF) conditions. Modulators of **(A)** action potential duration to 90% of repolarization (APD_90_), **(B)** Ca^2+^transient (CaT) duration to 80% of recovery (CaTD_80_), **(C)** CaT rise time (*t*_10-90_), and **(D)** Systolic peak of CaT. Parameter variability of 60%.

**Figure 2** represents the sensitivity of quantitative indicators of AP and CaT characteristics to changes in ion channels obtained in the univariate and multivariate sensitivity analyses. Sensitivities helped reveal the most important modulators of a determined electrophysiological property. High regression coefficients highlighted an important effect of a particular transport mechanism on Ca^2+^ indicators, taking into account the synergy between variables in the case of multivariate analysis. Our results showed that I_Kr_ was the parameter with the major impact on APD_90_ (**Figure 2A**), while SERCA had the leading role in Ca^2+^ biomarkers (**Figures 2B–D**). Sensitivities were normalized to allow comparison between the sensitivities obtained through a ± 60% univariate sensitivity analysis (light blue for N conditions and pink bars for HF conditions) with multivariate sensitivity analysis (dark blue for N conditions and red bars for HF conditions). In this comparison, results from both methodologies were consistent for APD sensitivities (**Figure 2A**). Small differences were though observed in the case of Ca^2+^ biomarkers (**Figures 2B–D**). For instance, **Figure 2B** shows that in HF, I_Kr_ seems to have a moderate impact on CaTD_80_ according to the univariate results (pink bars), while multivariate sensitivity indicates that the effect is lower (red bars). In other cases, with the univariate analysis, the relative importance of parameters can be altered between the N and HF conditions. This was the case for I_CaL_, I_NCX_, and J_leak_ on *t*_10-90_ (**Figure 2C** light blue and pink bars). **Figure 2D** shows how NCX has the main role in modulating systolic Ca^2+^ according to the univariate sensitivity analysis (light blue in N and pink bars in HF), while the multivariate sensitivity highlights SERCA and I_CaL_ as the main modulators in N (dark blue bars) and HF (red bar) conditions, respectively, and the exchanger has a secondary role.

Multivariate and univariate analyses also identified similar changes in parameter sensitivities from N to HF (**Figure 2, light and dark** blue vs pink and red), such as an increased impact of I_NaL_ on APD_90_ in HF with respect to normal conditions (**Figure 2A**), a higher I_Kr_ influence on CaTD_80_ (**Figure 2B**), and a decrease of SERCA modulation effect on systolic [Ca^2+^]_i_ (**Figure 2D**).

We further investigated the aforementioned discrepancies by performing additional sensitivity analyses with a lower conductance variability. Parameter variability was reduced to 15% in the univariate study and the standard deviation was decreased to 0.1 in the multivariate regression, resulting in sensitivities slightly different from those described above with higher variability (60% and σ = 0.3, respectively). After reducing parameter variability, negligible differences were found when comparing both methods (see **Supplementary Figures S1, S2**, light-colored bars). For example, considering low variability in HF (**Supplementary Figure S2D**, light bars), both analyses (univariate and multivariate) highlighted I_CaL_ as the most important parameter regulating systolic [Ca^2+^]_i_, while univariate sensitivities obtained with 60% variability (darker pink bar) highlighted I_NCX_ as the main regulator and underestimated the impact of I_CaL_.

Coefficients of determination of the multivariable regression are shown in **Table 2** for the different sensitivity analyses. Values were closer to 1 as variability decreased. CaTD_80_ and systolic [Ca^2+^]_i_ linear fit significantly improved with lower variability, especially in HF (0.938 vs 0.677 and 0.970 vs 0.792, respectively). This indicates that, with large parameter variability, and in the HF condition, significant non-linear relationships between parameter values and Ca^2+^ handling processes make the multivariable regression model less accurate.

**Table 2:** Coefficients of determination (R^2^) of the multivariable regression analyses in normal (N) and heart failure (HF).

*R*^2^	APD_90_	CaTD_80_	*t*_10-90_	[Ca^2+^]_i_ syst
N (σ = 0.3)	0.987	0.940	0.908	0.954
N (σ = 0.1)	0.994	0.997	0.975	0.994
HF (σ = 0.3)	0.987	0.677	0.871	0.792
HF (σ = 0.1)	0.999	0.938	0.910	0.970

In summary, univariate and multivariate sensitivity analyses yield very similar results for low variability of parameters, which would correspond to natural inter-subject electrophysiological differences. Univariate analysis is less computational expensive and is thus a valid methodology under these conditions. However, when large variability is applied, which would respond to effects of drugs, significant discrepancies arise between both methodologies. Multivariate analysis should be more reliable, at least for biomarkers with coefficients of determination close to one. Indeed, multivariate analysis considers interaction between the different parameters of the ion channels altered.

### Effects of Fibroblast–Myocyte Coupling in Normal and Failing Conditions

**FIGURE 3:**
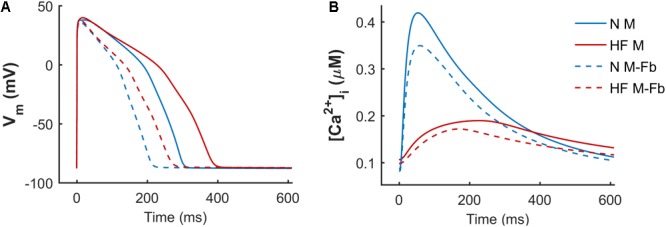
Effect of fibrosis on action potential **(A)** and Ca^2+^ transient **(B)**. Normal (N) and failing (HF) baseline myocyte models in an uncoupled myocyte (M) and a myocyte coupled to five fibroblasts (M-Fb).

When simulations were performed to electrically coupled fibroblasts to a myocyte, the myocyte’s AP and CaT were significantly changed. **Figure 3** shows APD and systolic [Ca^2+^]_i_ reduction in the normal baseline human endocardial model when fibroblasts were coupled (solid vs discontinuous blue trace), as well as in the failing baseline endocardial model (solid vs discontinuous red trace).

**FIGURE 4:**
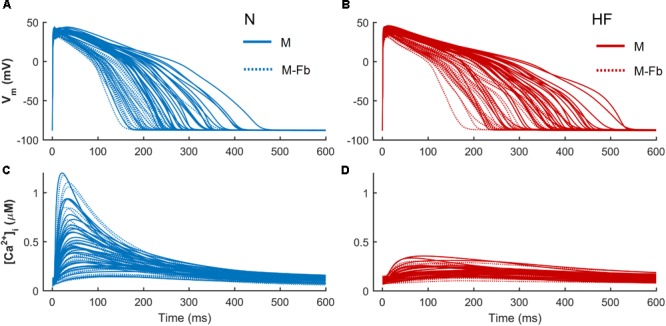
Action potential and Ca^2+^ transient samples (*n* = 30) of uncoupled myocytes (solid traces) and myocytes coupled to five fibroblasts (discontinuous traces) from two population of models, obtained from a multivariate set of ionic conductances (*n* = 300), using the basic ORdmm in normal conditions **(A,C)** and the basic HF remodeling model **(B,D)**.

When fibroblasts were coupled to myocytes from two populations of models (*n* = 300) generated for both N and HF conditions, the above-mentioned effects on APD and CaT were maintained, as shown in **Figure 4**. CaT traces in normal conditions (**Figure 4A**) with coupled fibroblasts (discontinuous blue traces) overlapped with CaT traces in the absence of fibroblasts (solid blue traces).

**FIGURE 5:**
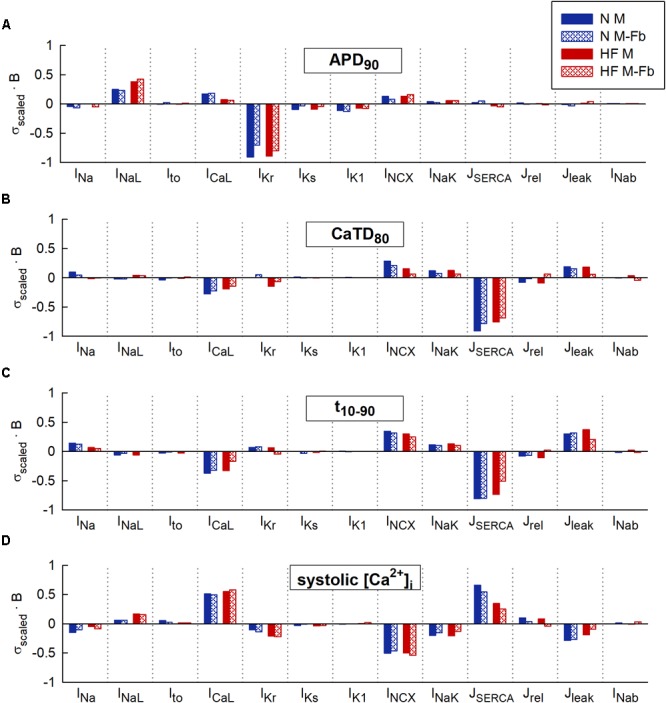
Comparison of sensitivities obtained from 4 multivariable regression analyses: normal (N) and heart failure (HF) conditions with or without coupled fibroblasts (Fb). Modulators of **(A)** action potential duration to 90% of repolarization (APD_90_), **(B)** Ca^2+^ transient (CaT) duration to 80% of recovery (CaTD_80_), **(C)** CaT rise time (*t*_10-90_), and **(D)** Systolic peak of CaT. High parameter variability. Regression coefficients (b) are scaled to the standard deviation (σ) of log-normal distributed biomarkers in uncoupled myocytes (M).

To evaluate whether fibroblasts coupling would significantly change sensitivities of electrophysiological biomarkers to parameters variability, multivariate analyses were conducted on these new populations. When a high variability of parameters was considered (σ = 0.3), our results showed that in the presence of fibroblasts, biomarkers sensitivities to ionic variables slightly changed with respect to uncoupled myocytes. As displayed in **Figure 5**, most of these differences were quantitative, as fibroblasts reduced sensitivities to parameters, but the qualitative role of each parameter was maintained. For instance, SERCA contribution to Ca^2+^ indicators (**Figures 5B–D**) decreased both in normal and failing conditions when fibroblasts were considered (discontinuous bars).

To confirm these results, we decreased parameter variability to σ = 0.1 and obtained more accurate regression coefficients. In the new range of parameter variability (see **Supplementary Figure S3**), sensitivities hardly changed with respect to higher variability.

### AP and CaT Restoration in HF

**FIGURE 6:**
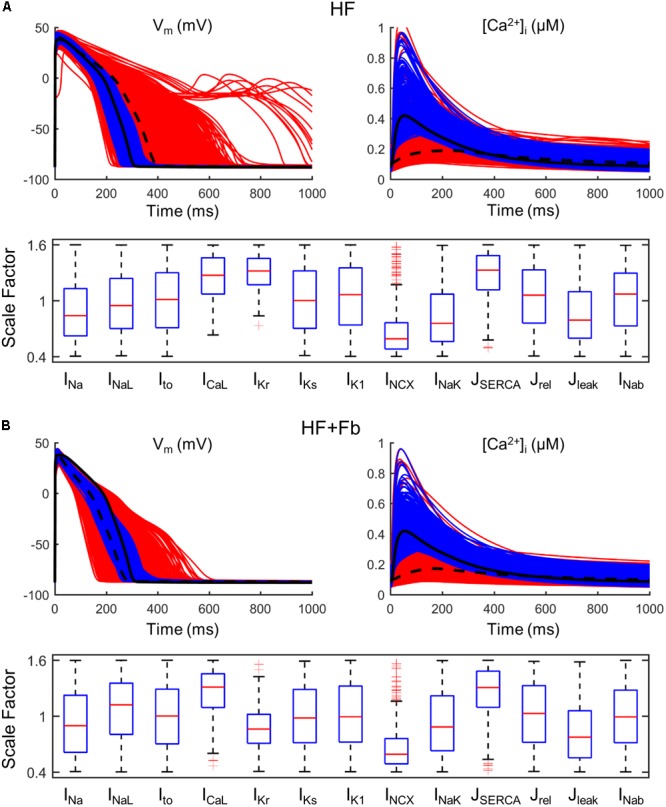
Restoration of normal action potential (AP) and calcium transient (CaT) in a population of failing models (*n* = 10,000). All simulated models (red traces) are obtained from a HF basic model (black dashed line) in which parameters have been varied ±60%. Calibrated models (blue traces) are within the limits of normal biomarkers (basic ORdmm in black solid line). Distributions of parameter scaling factors for the restored models. **(A)** Population of an uncoupled myocyte. **(B)** Population of a myocyte coupled to five fibroblasts.

To analyze how failing APs and CaTs could be restored and brought to normal ranges, a larger population of failing models was generated (*n* = 10,000). The population of models approach generates cells exhibiting a wide variety of physiological behaviors, including cells within the HF population that have APs and CaTs similar to those observed in healthy cells. Selecting this subpopulation and examining the distributions of parameters provides guidance for therapeutic targets. We identified parameter combinations restoring APs and CaTs to waveforms within a healthy range shown in **Table 1** in Section “Materials and Methods.” In **Figure 6A**, red traces represent all models generated from the baseline ORdmm HF model (black dashed line) and blue lines are models satisfying healthy ranges of both AP and CaT biomarkers. Electrophysiological HF phenotype was restored in around 500 models when I_Kr_, SERCA, and I_CaL_ activities were enhanced, and NCX function decreased. Interestingly, NCX distribution presented several outliers at high scaling factors. When myocytes were coupled to fibroblasts (**Figure 6B**), the role of SERCA, I_CaL_, and NCX was similar, whereas I_Kr_ modulation was not important.

### Mechanistic Analysis of Calcium Dynamics Impairment in Fibrosis

**FIGURE 7:**
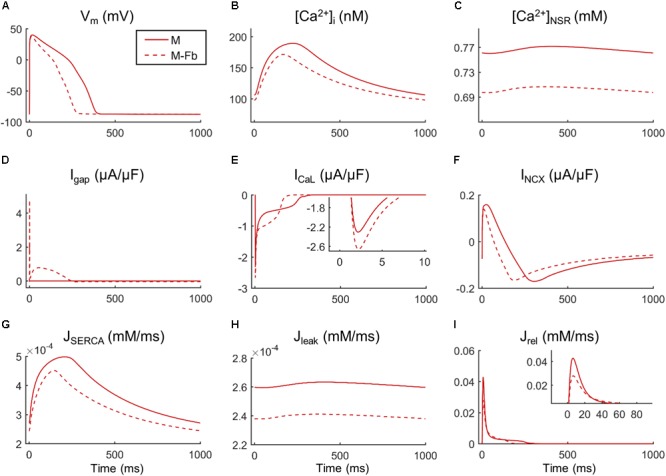
Main ionic properties and mechanisms in steady state of a failing myocyte interacting (M-Fb) or not (M) with fibroblasts. **(A)** Membrane potential (V_m_), **(B)** intracellular Ca^2+^ concentration ([Ca^2+^]_i_), **(C)** sarcoplasmic reticulum (SR) Ca^2+^ concentration ([Ca^2+^]_SR_), **(D)** outward positive current circulating from the myocyte to the fibroblast (I_gap_), **(E)** L-type Ca^2+^ current (I_CaL_), **(F)** Na^+^/Ca^2+^exchange current (I_NCX_), **(G)** SR Ca^2+^ uptake via SERCA pump (J_SERCA_), **(H)** SR Ca^2+^ leak (J_leak_), and **(I)** SR Ca^2+^ release flux via ryanodine receptors (J_rel_).

**FIGURE 8:**
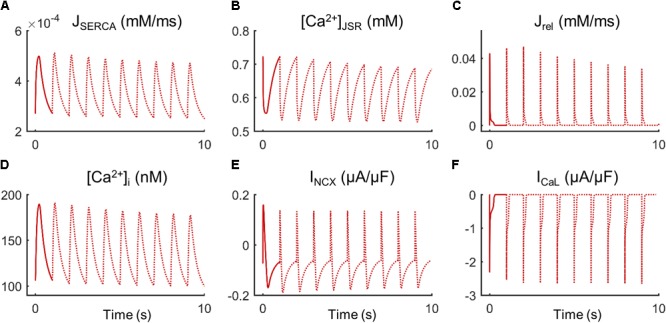
Transient state of ionic mechanisms and Ca^2+^ concentration in cellular compartments of a failing myocyte coupled to five fibroblasts (dashed lines). Initial steady state of an uncoupled myocyte (solid line). **(A)** SR Ca^2+^ uptake via SERCA pump (J_SERCA_), **(B)** junctional SR Ca^2+^ concentration ([Ca^2+^]_JSR_), **(C)** SR Ca^2+^ release flux via ryanodine receptors (J_rel_), **(D)** intracellular Ca^2+^ concentration ([Ca^2+^]_i_), **(E)** Na^+^/Ca^2+^exchange current (I_NCX_), and **(F)** L-type Ca^2+^ current (I_CaL_).

Fibroblast coupling decreased systolic Ca^2+^ in normal myocytes and exacerbated calcium dynamics impairment in failing myocytes, although parameter sensitivities were hardly affected. To understand the underlying mechanisms, ion currents were carefully analyzed in our simulations. **Figure 7** shows the traces of selected electrophysiological variables of the model in the time course of an AP at steady state for a failing myocyte with (solid line) and without (dashed line) fibroblast coupling. To understand how these steady-state conditions were reached, we analyzed transient changes in myocyte [Ca^2+^] after fibroblast coupling, as shown in **Figure 8**. In these panels, isolated myocytes are initially at steady state, then fibroblasts are coupled beginning on the second beat. As shown in **Figure 7C**, I_gap_ is an outward current flowing from the myocyte to fibroblasts, non-existing in the uncoupled myocyte. This outward current decreases membrane potential and has an indirect effect on voltage dependent currents such as I_CaL_. Indeed, we can observe that when fibroblasts are coupled (dashed traces) all currents and fluxes present a shorter duration, as well as AP, and a reduced peak (see, for instance, CaT in **Figure 7B**), except I_CaL_. Despite the reduced duration of this current, the initial I_CaL_ peak contributed to a larger Ca^2+^ influx through these channels (increase from 140 to 146 pC/μF). The integral of NCX was also computed and indicated an increase in inward NCX extruding more Ca^2+^ when fibroblasts were coupled (from 77 to 81 pC/μF). In the transient evolution shown in **Figure 8F**, the peak of I_CaL_ exhibited a maintained increase compared with the uncoupled myocyte (shown in solid red). Due to the increased Ca^2+^ extrusion through NCX, however, [Ca^2+^]_JSR_ progressively declined, leading to the decrease in [Ca^2+^]_i_ peak observed in steady state.

## Discussion

### Main Findings

In this study, two methodologies were used to perform sensitivity analyses evaluating the effects of fibroblast–myocyte coupling under normal and HF conditions. Our main findings are that (i) univariate and multivariate analyses yield very similar results and low variability of parameters yields more reliable multivariate sensitivity analyses, (ii) despite exacerbating Ca^2+^ impairment in HF, fibroblast to myocyte coupling does not alter the role of the main mechanisms regulating Ca^2+^ dynamics in myocytes, and (iii) drug action on I_CaL_ and SERCA enhancement and NCX block would help CaT restoration in HF regardless of fibrosis presence.

### Suitable Sensitivity Analyses

Univariate and multivariate sensitivity analyses were performed using an initial large variability (±60%) and then smaller (±15%). At lower variability, changes in electrophysiological properties behaved linearly, making results from univariate and multivariate analysis more similar. Indeed, ionic currents and fluxes work synergistically to generate AP and CaT. Variables such as membrane potential and ionic concentrations link all ionic mechanisms in a way that a change in one parameter (conductivity in this case) has an effect on other parameters, and final differences in electrophysiological properties are the result of an interaction of all these variations. In addition, within the range of variation of a specific parameter, the effects on biomarkers can be different, and assuming linearity can become less accurate when a wide interval is considered. In fact, as electrophysiological remodeling in HF is simulated by changing parameters, the same scale factor applied in N and HF implies a different range of variation of such parameters, accounting for different sensitivities to biomarkers. This explains the increase in APD sensitivity to I_NaL_, or the decrease of systolic [Ca^2+^]_i_ sensitivity to SERCA in HF with respect to N conditions.

Multivariate parameter sets, unlike varying one parameter at a time, can provide information about electrophysiological properties in a wide combination of parameters, which could be useful to evaluate the response of a drug in different individuals, instead of on a fixed baseline model. The high physiological or pathological variability of ionic parameters requires the analysis of the behavior in the whole range.

Despite accuracy loss with high parameter variability, sensitivity analyses are a useful systematic tool to determine the most important mechanisms involved in Ca^2+^ dynamics. We observed that univariate and multivariate sensitivities agreed in the most important parameters contributing to each biomarker. For instance, it was observed the strong impact of I_Kr_ on APD_90_, as O’Hara et al. (2011) described in their model. For CaT indicators, the main role of parameters such as SERCA, NCX, and I_CaL_ was highlighted with both methodologies, agreeing with experimental observations (Ozdemir et al., 2008; Rocchetti et al., 2008; Tamayo et al., 2017), and with a previous study comparing populations of normal and failing myocytes (Walmsley et al., 2013).

In electrophysiological models, biomarker sensitivities are usually calculated within a wide range of variation in ionic conductances, which has been reported experimentally. In their work, Romero et al. (2009) simulated univariate variations up to ±30% to evaluate the arrhythmogenic risk of ionic change, with AP properties falling within the experimental range. In the multivariable regression conducted by Sobie (2009), it was assessed how variability (σ ranging from 0.1 to 0.5) affected the regression model and observed a *R*^2^ reduction with the increase of σ, although many regression coefficients remained constant, being small the decrease in accuracy obtained for APD. This is consistent with our results, highlighting the robustness of sensitivity analysis for AP biomarkers. However, Ca^2+^-biomarkers sensitivities were more affected by variability. Comparing low and high parameter variability, sensitivities differed more in the univariate case due to changes in parameter–biomarker relation along the variation range, including non-linearities. Sensitivities derived from the multivariate study for HF showed the lowest coefficients of determination, suggesting that HF remodeling enhances non-linearities between variables.

In other modeling studies, experimentally-calibrated populations are first generated with conductances scaled up to ±100%. Constrained biomarkers according to experimental values determine the range of parameters in the subsequent regression analysis (Zhou et al., 2016; Britton et al., 2017). This way, linear fit problem could be solved but natural variability dominates the results and high effects of drugs are not evaluated in this range.

It can be concluded that the roles of the different parameters on electrophysiological biomarkers can be qualitatively estimated using linear methods, including different variability and parameter distributions. Furthermore, our findings highlight that both types of sensitivity studies, univariate and multivariate, provide similar results. However, when large variability is considered, discrepancies arising between these methodologies can become notable, affecting Ca^2+^ properties and patholological conditions to a greater extent. Univariate analysis is less computational expensive and is thus a valid methodology within a moderate variability range and for a reasonable number of parameters. Quantitative results from the multivariate analysis should be more reliable, as parameter interaction is considered, but caution should be taken if coefficients of determination are not close to one, which can happen under HF conditions.

### Fibrosis Exacerbates Ca^2+^ Transient Impairment in Heart Failure

This computational study shows that the electrical activity of myocytes, including calcium dynamics, is affected by fibroblast coupling. Specifically, CaT peaks are smaller and SR [Ca^2+^] is reduced when fibroblasts are coupled to myocytes. Despite these alterations, the ionic mechanisms regulating Ca^2+^ cycling are barely affected by the intercellular interaction, indicating that fibroblast coupling does not influence which pathways represent the best drug targets. HF remodeling seems to have a greater impact on the relative role of the different ionic mechanisms that regulate Ca^2+^ cycling. However, our results show that fibroblast coupling could be contributing to the excitation–contraction coupling impairment seen in HF.

An important effect observed when simulating fibroblast–myocyte coupling is the marked APD shortening. Experimental studies showing the existence of gap junctions (connexin43) between fibroblasts and myocytes also revealed changes in AP waveform and conduction velocity due to a modulation of myocyte electrophysiology (Gaudesius et al., 2003; Miragoli et al., 2006; Zlochiver et al., 2008). Previous computational studies exploring fibroblast–myocyte electrophysiological interactions also showed reductions in APD. When MacCannell et al. (2007) developed the mathematical model for the active fibroblast, they coupled it to a human myocyte model and reported an APD shortening, as fibroblasts acted as current sinks. The theoretical work of Jacquemet and Henriquez (2008) also showed that the effect of coupling caused a faster myocyte repolarization, but changing fibroblast properties, such as a less negative fibroblast resting potential, reversed the effect. It is known that fibroblasts differentiate into myofibroblasts in response to inflammation, an activated form which presents contractile proteins, implying the existence of Ca^2+^ cycling (Chilton et al., 2007). To date, no specific models for myofibroblasts have been developed but simulations have been performed increasing the membrane capacitance and depolarizing the resting membrane potential of the fibroblast model (Nguyen et al., 2012; Sridhar et al., 2017). In the present study, we used the fibroblast model by MacCannell et al. (2007) with a resting potential of -49.4 mV, thus the effect in normal and failing myocytes was a reduction in APD. We also conducted some additional simulations (see **Supplementary Figure S4**) changing to myofibroblast phenotype (C_F_ = 50 pF, E_F_ = -24.5 mV), and we observed similar effects, i.e., APD shortening and Ca^2+^-handling impairment. The improvement of myofibroblast models, incorporating Ca^2+^ dynamics, would certainly shed light into the understanding of Ca^2+^ dynamics alterations in the failing tissue.

The number of coupled fibroblasts considered could also alter the results. The uncertainty about the *in vivo* coupling, different degrees of fibrosis, and the difficult to quantify coupled fibroblasts in tissue has led to the exploration of different values of G_gap_ or a range of myocyte–fibroblast ratios (Jacquemet and Henriquez, 2008; Sachse et al., 2009; Xie et al., 2009a). We compared the effects of coupling one fibroblast to five (results not shown) and we found that the impact on APD and CaT was lower with one fibroblast and increasing the number of fibroblasts to five did not increase the effects fivefold. This finding suggested that the sensitivity of the myocyte to G_gap_ is not linear, saturating for higher values, as observed in other analyses of G_gap_ effects when strong coupling was considered (Jacquemet and Henriquez, 2008). We finally used five fibroblasts in order to represent fibroblast proliferation observed in pathological conditions.

In our simulations, the baseline ORdmm model showed a notable Ca^2+^ impairment when fibroblast interaction was considered in both N and HF conditions, but the population of models revealed that not every individual might have an impaired Ca^2+^ handling in a fibrotic heart. However, in HF, even a minor Ca^2+^-handling alteration should be considered important because it worsens contractility dysfunction. Only a few studies have measured intracellular Ca^2+^ in myocytes interacting with fibroblasts. In co-culture experiments used to investigate the crosstalk between both cell types, it was found that fibroblasts from normal hearts increased CaT amplitude, while fibroblasts and myofibroblasts from pressure-overloaded hearts led to a smaller amplitude associated with a reduction in SR Ca^2+^ content (Cartledge et al., 2015). A more recent *in vitro* study to explore the influence of heterocellular interactions on cardiomyocyte function showed that only adult fibroblasts had significant consequences on the electrical and mechanical function, by prolonging APD and reducing CaT amplitude (Li et al., 2017). In computational studies, only the work by Zhan et al. (2014) has considered the role of fibroblast proliferation in a mathematical model with Ca^2+^, and reported a longer APD and CaT width, modulating cardiac electromechanical behavior. This discrepancy with our results, a prediction of APD prolongation rather than shortening, might be due to the use of a passive fibroblast model with a resting membrane potential set to -20 mV. Indeed, when modeling fibroblast electrophysiological behavior, the values of several parameters are crucial in the outcome, including the number of fibroblasts coupled to each myocyte, the resting potential, and the capacitance of each fibroblast (Chilton et al., 2005), and whether myofibroblast properties are considered (Sridhar et al., 2017).

Our sensitivity analysis showed minimal differences in the mechanisms determining APD and CaT waveform between myocytes coupled or not to fibroblasts. This could simplify therapies, as presence or absence of fibrotic tissue would not alter the treatment. The slightly reduced sensitivities in the presence of fibrosis indicate that targeted ion transport pathways require a higher variation to produce the same percentage of change to improve the considered electrophysiological property. I_Kr_ is the main modulator of APD, as O’Hara et al. (2011) reported in their sensitivity analysis, and its enhancement in HF would restore the prolonged APD. However, in myocytes coupled to fibroblasts there is no need to reduce APD via I_Kr_ modulation as fibroblasts act as a current sink and lead to an earlier repolarization. Regarding CaT indicators, HF remodeling seems to be the main cause of Ca^2+^ alteration, which explains why coupled and uncoupled myocytes require the same measures to restore Ca^2+^ cycling: increase of SERCA and I_CaL_, and NCX reduction.

Finally, the mechanistic analysis of Ca^2+^ cycling with fibroblasts reveals that, despite an increase in I_CaL_ peak with fibroblast coupling, increased NCX Ca^2+^ extrusion leads lower SR content and smaller CaT peaks. In the steady state, there is a balance between fluxes, maintaining constant Ca^2+^ levels, which does not happen in the transient state. In fact, the progressive reduction in intracellular Ca^2+^ is a sign of negative balance between Ca^2+^ influx and efflux in the myocyte, which occurs after a perturbation in the system, i.e., fibroblast coupling. The modulation of all ionic mechanisms including Ca^2+^ cycling is caused by the active role of fibroblasts, becoming a current sink. The new outward current in myocytes, I_gap_, accelerated AP repolarization and consequently, all voltage-dependent currents were affected, including I_CaL_. The model used for human endocardial AP does not present a notch with a marked early repolarization phase as in other species. For this reason, I_gap_ does not affect the excitation–contraction coupling by changing the rate of the initial repolarization as in Xie et al. (2009b). In the present study, the implicated mechanism becomes more important in later phases of the AP. We also conducted simulations using the epicardial model of ventricular AP formulated by O’Hara et al. (2011) to see the effect on the early repolarization phase which is indeed present in the epicardial model. As shown in **Supplementary Figure S5**, no important changes are observed in this phase under the effect of coupled fibroblasts.

The static formulation of myocyte–fibroblast coupling differed from the dynamic gap junctions channels modeled by Brown et al. (2016). They observed a reduction in the junctional current during the upstroke of the AP when considering time- and voltage-sensitive gating channels in homotypic and heterotypic channels, i.e., with different connexins combinations (Cx43 and Cx45). Although it did not significantly alter conduction velocity compared to static gap junctions, fibroblasts with a smaller sink impact could have a different effect on Ca^2+^ dynamics.

Our findings showed that a higher I_CaL_ peak introduced more Ca^2+^ in the myocyte but did not trigger a higher SR Ca^2+^ release as expected. According to Shannon et al. (2000), there cannot be a release from the SR with a Ca^2+^ load of less than 50% of its maximal content, explaining that the reduced SR Ca^2+^ content characteristic of failing myocytes contributes to reduce force development. Similarly, changes in SR Ca^2+^ load of 58% have been measured in failing isolated myocytes, and related to a smaller CaT (Piacentino et al., 2003). Therefore, a reduction in SR Ca^2+^ content due to fibroblasts could be the cause of an exacerbated Ca^2+^ impairment. The transient evolution shown in **Figure 8** helps understand the mechanisms leading to cellular Ca^2+^ loss. One of the advantages of mathematical models is the power to analyze hypothetical situations that cannot be measured experimentally, such as myocyte response to a sudden electrical connection with fibroblasts. In the transient state (**Figure 8**), we observed that I_CaL_ peak increased since the first beat, due to the immediate effects of I_gap_ on AP. Initially, there was also an intracellular Ca^2+^ rise, as SR Ca^2+^ content was still elevated. The mechanisms to remove Ca^2+^ from the cytosol were then activated: SERCA and inward current through NCX (extruding Ca^2+^). NCX role, extruding Ca^2+^ out of the cell, becomes relevant in the transient state because if there is an imbalance with Ca^2+^ influx through I_CaL_ channels, Ca^2+^ loss occurs. This mechanism might explain the reduced SR Ca^2+^ load in myocytes interacting with fibroblasts.

Our findings suggest that fibroblasts increase Ca^2+^ impairment in HF by further reducing SR Ca^2+^ content in myocytes.

### Limitations

Several limitations need to be considered when drawing conclusions from the present study. Although simulations of cellular electrophysiological behavior and systematic analyses of biomarkers complement and enrich experimental research, uncertainties in the development of the mathematical models employed might affect the outcome of the simulations. The limited availability of electrophysiology data from human fibroblasts has led to the use of a fibroblast model developed on the basis of adult rat ventricular tissue data, without taking into consideration potential changes in HF. Fibroblasts with the same characteristics were considered in normal and failing conditions, but electrophysiological remodeling in fibroblast currents could have an additional effect on myocytes (Aguilar et al., 2014). Another limitation is the use of a fibroblast model, instead of using a myofibroblast model (not available to date), which is the characteristic form in pathological conditions, which would take into account Ca^2+^ dynamics in these cells. Ca^2+^ signaling has been observed in human cardiac fibroblasts although with different pathways to those in contractile myocytes (Chen et al., 2010), and myofibroblasts show intracellular CaTs, modulated by intercellular coupling with myocytes (Chilton et al., 2007), but neither of them have been modeled. Specific data for fibroblasts features are still lacking and one of the major concerns in studies focusing myocyte–fibroblast interactions is the use of data from co-cultures and the need of data describing the real electrophysiological behavior *in vivo*.

Gap junctional coupling between a myocyte and a fibroblast has been modeled by a simple conductance as in previous simulation studies of fibroblast-myocyte coupling. However, recent findings suggesting the interaction of connexin45 with CaM (Zou et al., 2017) highlight that some ions such as Ca^2+^ can modulate the coupling current. If the modulation of the intercellular coupling by Ca^2+^ ions was taken into account, larger alterations in Ca^2+^ dynamics mechanisms could be observed. Any mechanism related to Ca^2+^ included into our model, such as realistic Ca^2+^ dynamics in fibroblasts, may affect the regulation of Ca^2+^ cycling in myocytes, and enhance the influence of fibroblasts.

Other structural modifications in HF, concerning myocytes, involve the loss of transverse tubules and reorganization of the cell membrane (Lyon et al., 2009). We have assumed that with the electrophysiological remodeling applied to ionic mechanisms, we qualitatively reproduce the delayed SR Ca^2+^ release resulting from detubulation. However, a detailed model including local changes in membrane structure related to the spatial organization of ion channels in HF could provide more accurate results about Ca^2+^ impairment (Nivala et al., 2015; Sanchez-Alonso et al., 2016).

Although Ca^2+^ homeostasis is related to contraction and relaxation force, other mechanical factors, such as myofibroblast contractility when fibroblasts are differentiated into the activated form or extracellular stiffness due to the excessive accumulation of collagen in HF, can contribute to cardiac dysfunction. Our model only considers intracellular Ca^2+^ in myocytes at a cellular level to evaluate the contraction of the heart. The behavior in tissue or even in the whole organ could also be different. A defined architecture of the myocyte when considering spatial distribution would allow to take into consideration the distribution of gap junctions as well as the extracellular space which could modulate Ca^2+^ homeostasis as it does in electrical propagation (Cabo and Boyden, 2009; Sachse et al., 2009; Seidel et al., 2010; Greisas and Zlochiver, 2016). We consider a natural continuation of the present work the analysis of fibrosis effects on calcium waves in 2D and 3D tissues, in which altered impulse propagation and generation of arrhythmias should develop because of the heterogeneities in tissue, according to other studies (Zlochiver et al., 2008; Sachse et al., 2009; Nguyen et al., 2012; Gomez et al., 2014a; Greisas and Zlochiver, 2016; Zimik and Pandit, 2016).

Regarding sensitivity analyses, the non-linearities are the main issue in quantifying the impact of parameters on biomarkers. To restore the electrical activity and contraction of myocytes, the effect of some drugs can imply a high change in an ionic mechanism, and the predictive value of this methodology decreases with increased variability.

Nonetheless, despite these limitations, cellular simulations can shed light in the causes of Ca^2+^ impairment observed in HF, which together with the arrhythmogenic activity of fibrotic tissue, can compromise the function of the myocardium. In the present study, the measures suggested to restore Ca^2+^ dynamics and contractility at cellular level are reliable because they are supported by experiments and according to our findings, tackling the electrophysiology remodeling in failing myocytes can also improve the effects of fibroblasts interactions.

## Author Contributions

MM, JF, and BT conceived and designed the study, and analyzed the data. MM performed simulations. JG and ES contributed to the methodology. MM and BT wrote the first draft of the manuscript. All authors contributed to manuscript revision, read, and approved the submitted version.

## Conflict of Interest Statement

The authors declare that the research was conducted in the absence of any commercial or financial relationships that could be construed as a potential conflict of interest.
