# Supplementary material for: Ca2+ Cycling Impairment in Heart Failure Is Exacerbated by Fibrosis: Insights Gained From Mechanistic Simulations
Source: Front Physiol. 2018 Aug 23;9:1194. doi: 10.3389/fphys.2018.01194 (PMC6116328; doi:10.3389/fphys.2018.01194)
Supplement: Supplementary file 1 [file Data_Sheet_1.pdf]

Supplementary Material

Ca^{2+} Cycling Impairment in Heart Failure is Exacerbated by Fibrosis: Insights Gained from Mechanistic Simulations

Maria T Mora, Jose M Ferrero, Juan F Gomez, Eric A Sobie, Beatriz Trenor*

*** Correspondence:** Beatriz Trenor: btrenor@eln.upv.es

In the following tables we provide the required information to replicate our simulations:

Supplementary Table 1. Changes in ORd model leading to ORdmm

Ion current	New formulation	References
I_{Na}	$G_{Na} = 31$ mS/µF $h_{ss} = j_{ss} = \frac{1}{1 + e^{\frac{V+78.5}{6.22}}}$ $h_{ssp} = \frac{1}{1 + e^{\frac{V+84.7}{6.22}}}$ $m_{ss} = \frac{1}{1 + e^{-\frac{V+48.97}{7.5}}}$	(Mora et al., 2017; Passini et al., 2016; ten Tusscher et al., 2004)
I_{NaL}	$G_{NaL} = 0.0144$ mS/µF	(Maltsev and Undrovinas, 2006; Mora et al., 2017)

G_{Na} and G_{NaL} are maximal conductances of fast (I_{Na}) and late Na^+ currents (I_{NaL}), V is membrane potential and h_{ss}, j_{ss}, h_{ssp} and m_{ss} are steady-state inactivation and activation gates of I_{Na}.

Supplementary Table 2. Heart failure (HF) remodeling in ORdmm model.

**Ionic parameter**	**% of change compared to the ORdmm**	**Experimental references**
I_{NaL}	180 %	(Maltsev et al., 2007)
τ_{hL}	180 %	(Maltsev et al., 2007)
I_{to}	40 %	(Beuckelmann et al., 1993)
I_{K1}	68 %	(Tomaselli and Marbán, 1999)
I_{NaK}	70 %	(Bundgaard and Kjeldsen, 1996; Tomaselli, 2004; Tomaselli and Marbán, 1999)
I_{NCX}	175 %	(Winslow et al., 1999)
CaMKa	150 %	(Antoons et al., 2007; Sossalla et al., 2011)
J_{SERCA}	50 %	(Piacentino et al., 2003)
J_{leak}	130 %	(Bers et al., 2006)
$K_{rel,Ca}$	80 %	(George, 2007)

The modified parameters are the maximum values of the late Na^+ current (I_{NaL}), the time constant of inactivation of the I_{NaL} (τ_{hL}), the transient outward current (I_{to}), the inward rectifier K^+ current (I_{K1}), the Na^+/K^+ pump current (I_{NaK}), the Na^+/Ca^{2+} exchanger (I_{NCX}), the fraction of active binding sites of the Ca^{+2} calmodulin-dependent protein kinase II (CaMKa), the sarcoplasmic reticulum (SR) Ca^{2+} pump (J_{SERCA}), the SR Ca^{2+} leak (J_{leak}) and the sensitivity to $[Ca^{2+}]_{JSR}$ of the ryanodine receptors (Ca^{2+} sensitivity of $J_{rel,\infty}$, called $K_{rel,Ca}$).

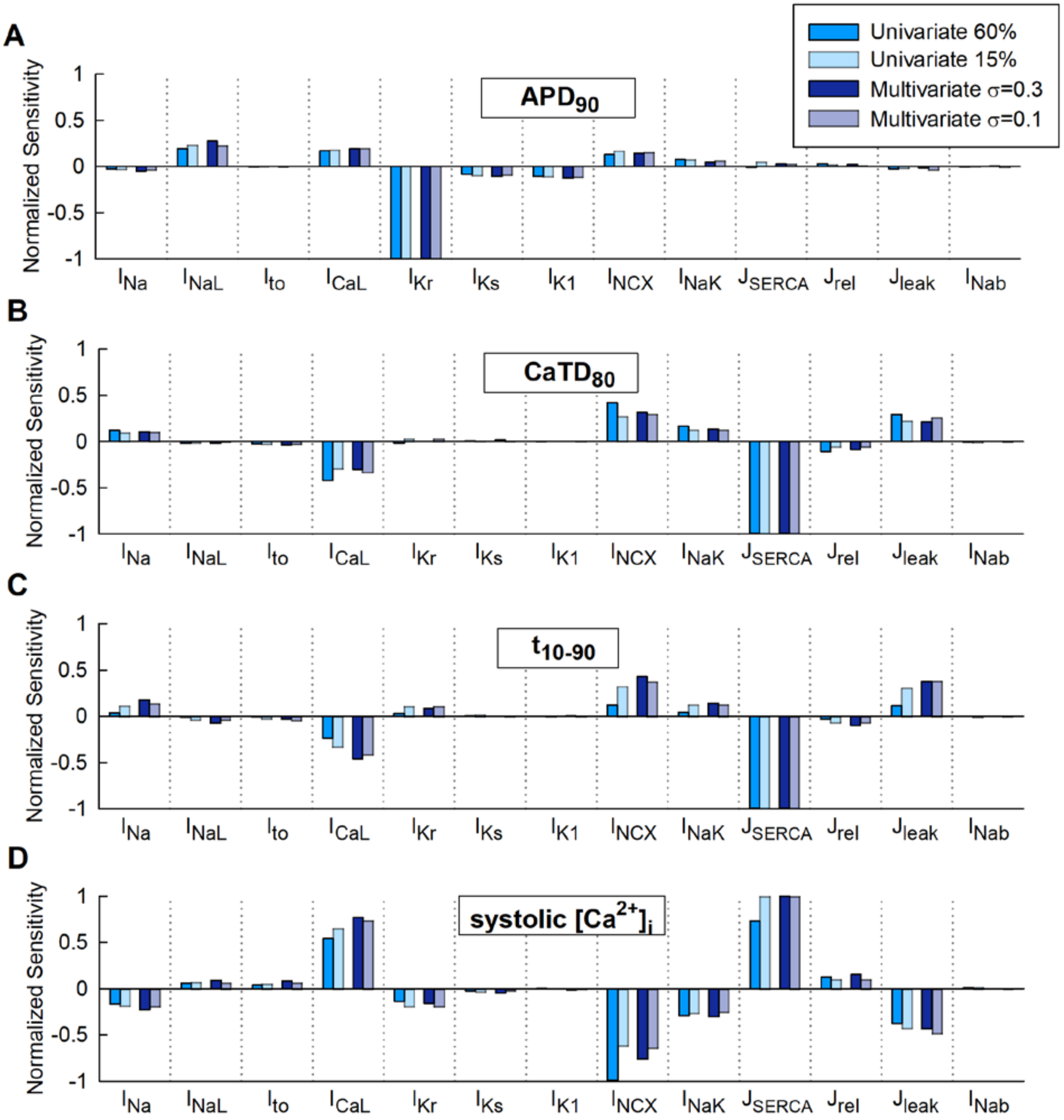

Supplementary Figure 1. Univariate and multivariate sensitivities in normal conditions with high and low parameter variability. Modulators of A) action potential duration to 90% of repolarization (APD_{90}), B) Ca^{2+} transient (CaT) duration to 80% of recovery ($CaTD_{80}$), C) CaT rise time ($t_{10\text{-}90}$) and, D) Systolic peak of CaT.

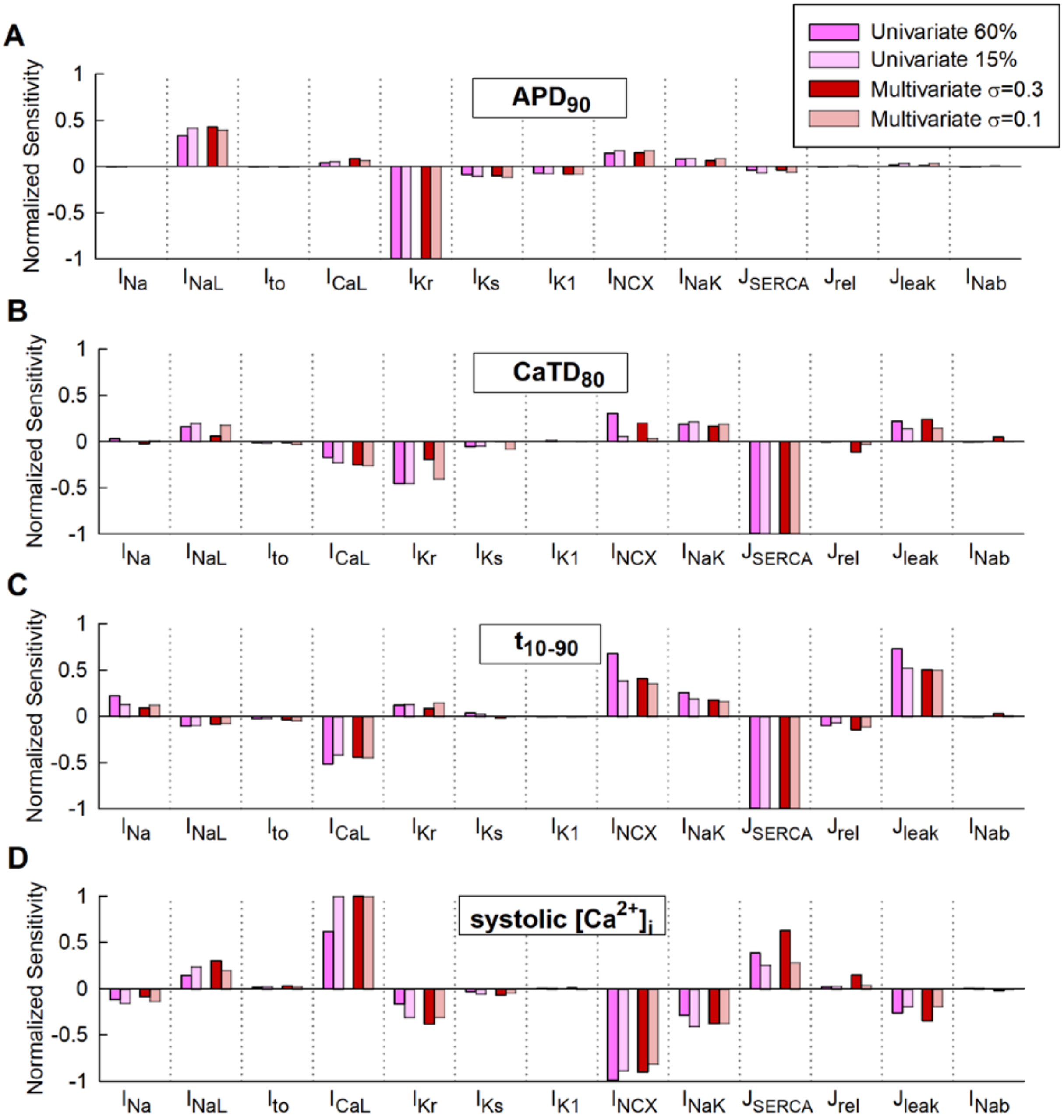

Supplementary Figure 2. Univariate and multivariate sensitivities in heart failure with high and low parameter variability. Modulators of A) action potential duration to 90% of repolarization (APD_{90}), B) Ca^{2+} transient (CaT) duration to 80% of recovery ($CaTD_{80}$), C) CaT rise time ($t_{10\text{-}90}$) and, D) Systolic peak of CaT.

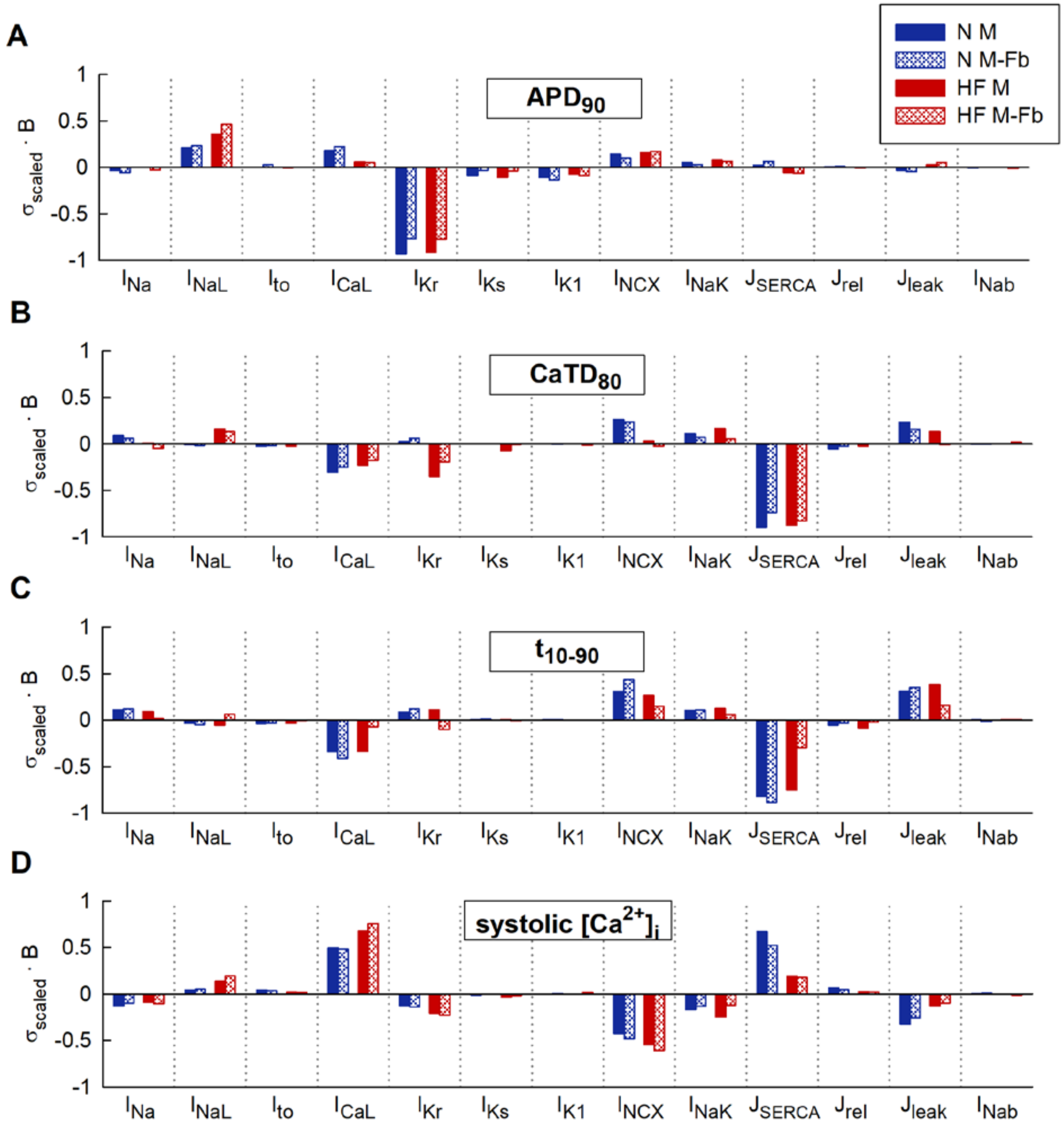

Supplementary Figure 3. Comparison of sensitivities obtained from 4 multivariable regression analyses: normal (N) and heart failure (HF) conditions with or without coupled fibroblasts (Fb). Modulators of A) action potential duration to 90% of repolarization (APD_{90}), B) Ca^{2+} transient (CaT) duration to 80% of recovery ($CaTD_{80}$), C) CaT rise time ($t_{10\text{-}90}$) and, D) Systolic peak of CaT. Low parameter variability (σ=0.1). Regression coefficients (B) are scaled to the standard deviation (σ) of log-normal distributed biomarkers in uncoupled myocytes (M).

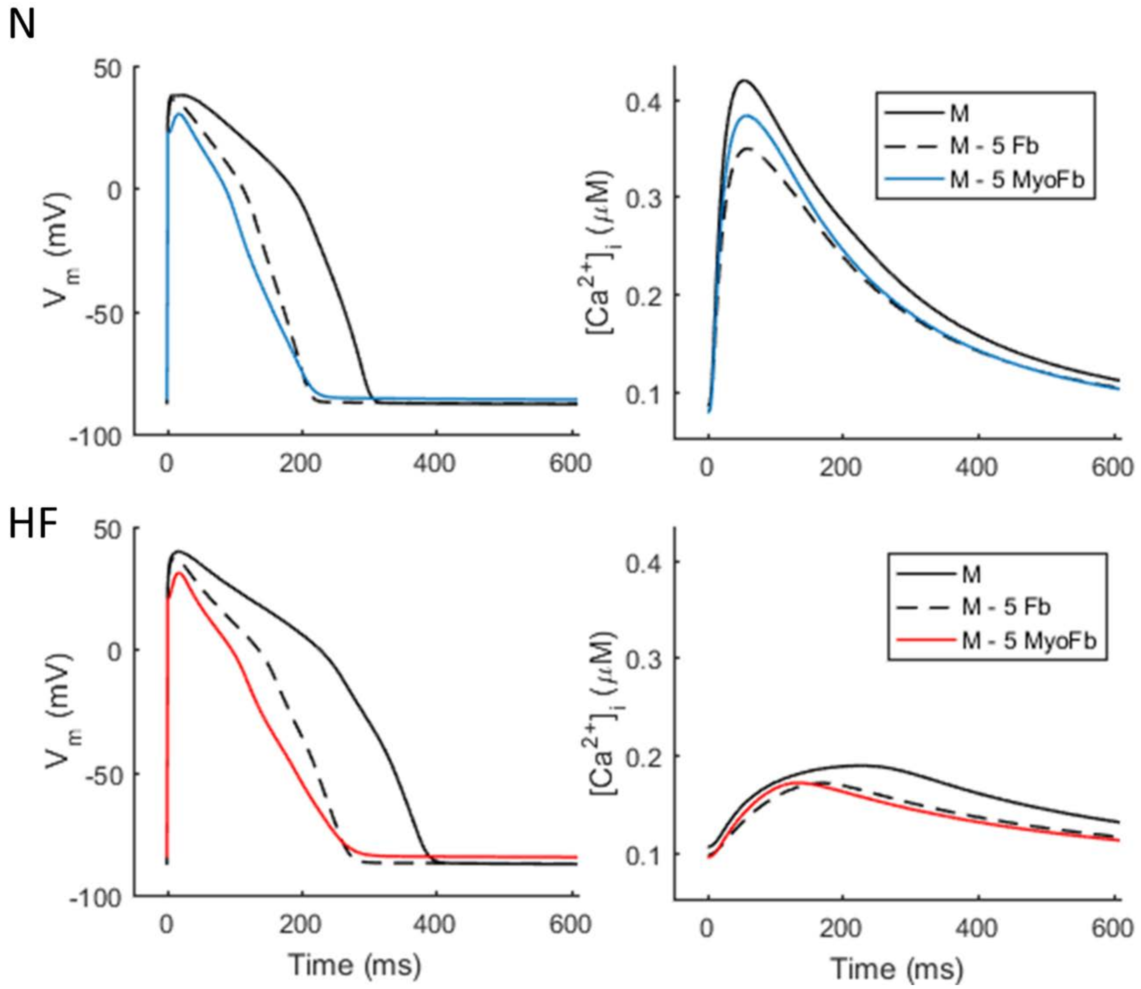

Supplementary Figure 4. Comparison of the effects of coupling fibroblasts (Fb) and myofibroblasts (MyoFb) on action potential and Ca^{2+} transient of a myocyte (M) in normal (N) and heart failure (HF) conditions. For myofibroblast simulations, MacCannell et al. (2007) active model was modified, increasing C_m to 50 pF and depolarizing the resting membrane potential of the fibroblast to -24.5 mV.

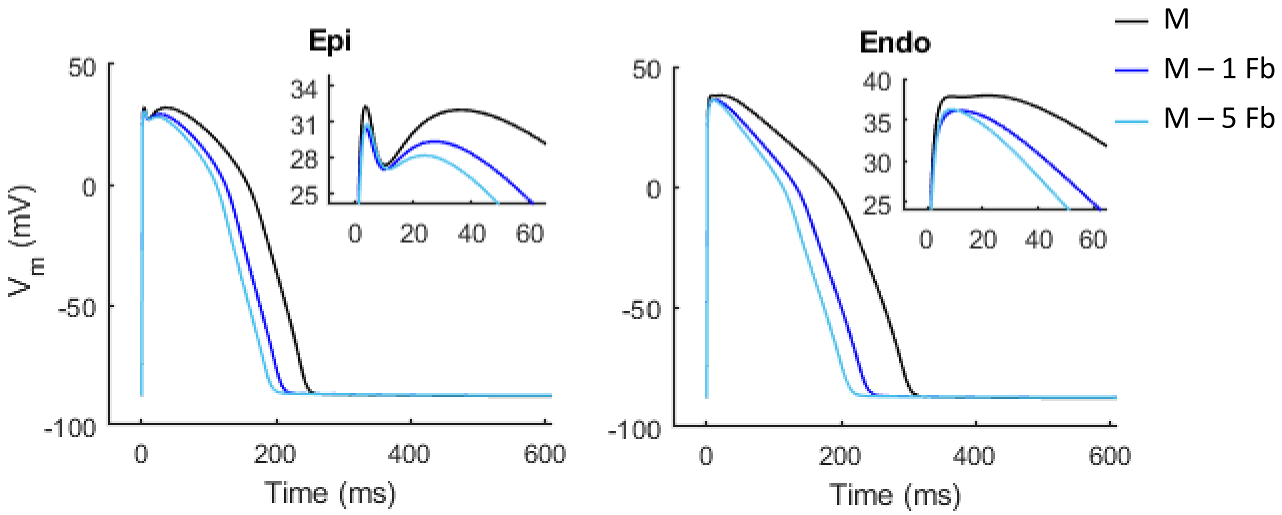

Supplementary Figure 5. Comparison of the effects of coupling fibroblasts (Fb) to epicardial and endocardial cells (M) on action potential waveform. Insets show phase 1 with higher resolution. The number of coupled fibroblasts is 0 (M), 1 and 5.

REFERENCES

Antoons, G., Oros, A., Bito, V., Sipido, K. R., and Vos, M. A. (2007). Cellular basis for triggered ventricular arrhythmias that occur in the setting of compensated hypertrophy and heart failure: considerations for diagnosis and treatment. *J. Electrocardiol.* 40, S8–S14. doi:10.1016/j.jelectrocard.2007.05.022.

Bers, D. M., Despa, S., and Bossuyt, J. (2006). Regulation of Ca2+ and Na+ in Normal and Failing Cardiac Myocytes. *Ann. N. Y. Acad. Sci.* 1080, 165–177. doi:10.1196/annals.1380.015.

Beuckelmann, D. J., Nabauer, M., and Erdmann, E. (1993). Alterations of K+ currents in isolated human ventricular myocytes from patients with terminal heart failure. *Circ. Res.* 73, 379–385. doi:10.1161/01.RES.73.2.379.

Bundgaard, H., and Kjeldsen, K. (1996). Human myocardial Na,K-ATPase concentration in heart failure. *Mol. Cell. Biochem.* 163–164, 277–283. doi:10.1007/BF00408668.

George, C. H. (2007). Sarcoplasmic reticulum Ca2+ leak in heart failure: mere observation or functional relevance? *Cardiovasc. Res.* 77, 302–314. doi:10.1093/cvr/cvm006.

MacCannell, K. A., Bazzazi, H., Chilton, L., Shibukawa, Y., Clark, R. B., and Giles, W. R. (2007). A mathematical model of electrotonic interactions between ventricular myocytes and fibroblasts. *Biophys. J.* 92, 4121–4132. doi:10.1529/biophysj.106.101410.

Maltsev, V. A., Silverman, N., Sabbah, H. N., and Undrovinas, A. I. (2007). Chronic heart failure slows late sodium current in human and canine ventricular myocytes: Implications for repolarization variability. *Eur. J. Heart Fail.* 9, 219–227. doi:10.1016/j.ejheart.2006.08.007.

Maltsev, V. A., and Undrovinas, A. I. (2006). A multi-modal composition of the late Na+ current in human ventricular cardiomyocytes. *Cardiovasc. Res.* 69, 116–127. doi:10.1016/j.cardiores.2005.08.015.

Mora, M. T., Ferrero, J. M., Romero, L., and Trenor, B. (2017). Sensitivity analysis revealing the effect of modulating ionic mechanisms on calcium dynamics in simulated human heart failure. *PLoS One* 12, e0187739. doi:10.1371/journal.pone.0187739.

Passini, E., Mincholé, A., Coppini, R., Cerbai, E., Rodriguez, B., Severi, S., et al. (2016). Mechanisms of pro-arrhythmic abnormalities in ventricular repolarisation and anti-arrhythmic therapies in human hypertrophic cardiomyopathy. *J. Mol. Cell. Cardiol.* 96, 72–81. doi:10.1016/j.yjmcc.2015.09.003.

Piacentino, V., Weber, C. R., Chen, X., Weisser-Thomas, J., Margulies, K. B., Bers, D. M., et al. (2003). Cellular basis of abnormal calcium transients of failing human ventricular myocytes. *Circ. Res.* 92, 651–658. doi:10.1161/01.RES.0000062469.83985.9B.

Sossalla, S., Maurer, U., Schotola, H., Hartmann, N., Didié, M., Zimmermann, W.-H., et al. (2011). Diastolic dysfunction and arrhythmias caused by overexpression of CaMKIIδC can be reversed by inhibition of late Na+ current. *Basic Res. Cardiol.* 106, 263–272. doi:10.1007/s00395-010-0136-x.

ten Tusscher, K. H. W. J., Noble, D., Noble, P. J., and Panfilov, A. V (2004). A model for human ventricular tissue. *Am. J. Physiol. Heart Circ. Physiol.* 286, H1573-89. doi:10.1152/ajpheart.00794.2003.

Tomaselli, G. F. (2004). What Causes Sudden Death in Heart Failure? *Circ. Res.* 95, 754–763. doi:10.1161/01.RES.0000145047.14691.db.

Tomaselli, G., and Marbán, E. (1999). Electrophysiological remodeling in hypertrophy and heart failure. *Cardiovasc. Res.* 42, 270–283. doi:10.1016/S0008-6363(99)00017-6.

Winslow, R. L., Rice, J., Jafri, S., Marban, E., and O'Rourke, B. (1999). Mechanisms of Altered Excitation-Contraction Coupling in Canine Tachycardia-Induced Heart Failure, II : Model Studies. *Circ. Res.* 84, 571–586. doi:10.1161/01.RES.84.5.571.
